# Incorporation of potassium halides in the mechanosynthesis of inorganic perovskites: feasibility and limitations of ion-replacement and trap passivation†

**DOI:** 10.1039/c8ra08823c

**Published:** 2018-12-12

**Authors:** Yousra El Ajjouri, Vladimir S. Chirvony, Michele Sessolo, Francisco Palazon, Henk J. Bolink

**Affiliations:** Instituto de Ciencia Molecular, ICMol, Universidad de Valencia C/ Catedrático J. Beltrán 2 46980 Paterna Spain Francisco.Palazon@uv.es; UMDO (Unidad de Materiales y Dispositivos Optoelectrónicos), Instituto de Ciencia de los Materiales, Universidad de Valencia Valencia 46071 Spain

## Abstract

Potassium halides (KX; X = I, Br, or Cl) were incorporated as partial replacements of CsBr in the mechanosynthesis of CsPbBr_3_. This led to partial substitution of both monovalent ions forming mixed Cs_1−*x*_K_*x*_PbBr_3−*y*_X_*y*_ perovskites. Longer photoluminescence lifetimes were also observed, possibly linked to the formation of a non-perovskite KPb_2_X_5_ passivating layer.

## Abstract 2

Potassium halides are used for cation-exchange, anion-exchange and trap passivation of mechanosynthesized perovskites.

## Graphical abstract

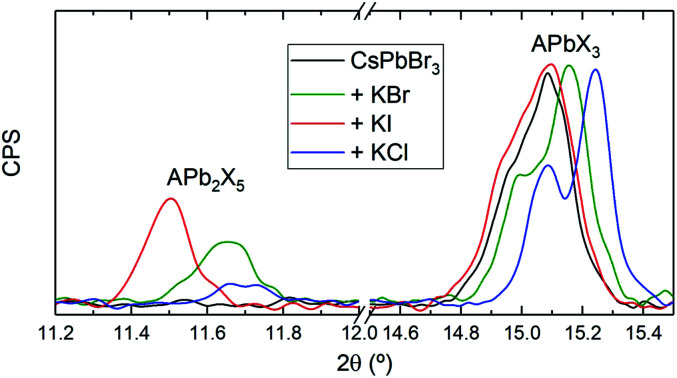

In the past few years, organic metal halide perovskites (OHPs) have drawn considerable attention as promising materials for optoelectronic devices.^1–5^ However, it is generally known that these materials make the development of stable solar cells and light emitting diodes rather difficult, due to their environmental instability related with the use of organic compounds.^6–8^ Thus, fully inorganic halide perovskites, such as cesium-based perovskites are sought after for their increased stability.^9–13^ The known poor solubility of cesium halides in common solvents may be bypassed by synthesizing inorganic perovskites in an all-dry manner such as by mechanosynthesis (*e.g.*, grinding or ball-milling)^14–18^ and/or thermal vacuum deposition.^19^ Recently, halide perovskites with an increasing complexity in formulation containing up to 6 or 7 different ions have proven to be beneficial for device performance.^20–24^ In this context, mechanosynthesis by ball-milling represents an ideal platform to test different precursors or additives in a simple manner. Multi-cation perovskites enable tuning of the bandgap of the material^23^ as well as its Goldschmidt tolerance factor (*t*),^25,26^ which is calculated as follows:
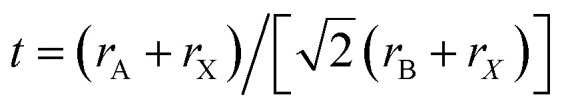
where *r*_A_, *r*_B_ and *r*_X_ respectively stand for the ionic radiuses of the cation A, metal B and the anion X in the ABX_3_ perovskite.

To obtain a stable cubic ABX_3_ perovskite, it is generally accepted that the Goldschmidt tolerance factor should not be lower than 0.8 nor exceed a value of 1. A *t*-value outside of this range usually results in non-perovskite structures. Examples of such crystalline structures are orthorhombic (so-called “yellow phase”) cesium lead iodide (CsPbI_3_) and formamidinium lead iodide (FAPbI_3_). In the case of CsPbI_3_, the tolerance factor is too small whereas in the case of FAPbI_3_ the tolerance factor is too large to result in a stable cubic phase at room temperature. However, the multi-cation cesium formamidinium lead iodide perovskite ((Cs:FA)PbI_3_) was shown to be stable.^23,27^ This is only an example of the interest of multi-cation perovskites. Among other cations, potassium has been recently used as an additive in perovskites, with different conclusions.^22,28–33^ Some reports show a benefit from the presence of potassium in mixed (KCs)PbI_3_, where the guest cation is capable of stabilizing the perovskite structure.^29^ Others, based on the small tolerance factor of such structure, have concluded that incorporating potassium halides in the synthesis does not lead to the effective incorporation of potassium as replacement of the “A” cation within the perovskite structure. As a result, potassium stays at the grain boundaries and indirectly contributes to surface passivation by providing additional halides (bound to K^+^), partially compensating the halide vacancies. The halide vacancies are believed to be one of the main quenching traps which need to be passivated to improve the optoelectronic properties of the perovskite.^28,34^ On the contrary, other reports have concluded that addition of potassium halides leads to the formation of different separate phases.^30,31^ These discrepancies might originate from the different perovskite crystallization processes used, which can result in different morphology, phase purity or stoichiometry of the final compound. Therefore, dry mechanosynthesis is an ideal preparation method, as it does not involve solvents, it avoids the formation of intermediate species, and eliminates the need of thermal treatments to foster the perovskite crystallization.

In this work we synthesized halide perovskites by ball milling equimolar mixtures of PbBr_2_ and ABr, where A = K_0.2_Cs_0.8_ and compared the resulting powders with the pure cesium reference (A = Cs). High resolution X-ray diffraction (XRD) patterns as well as optical characterization are presented in Fig. 1. The main diffraction peaks from the resulting powder are presented in Fig. 1b, c and e. These peaks correspond to the orthorhombic APbBr_3_ perovskite (see Fig. S1† for the full diffractograms and reference pattern ICSD 97851). Hence, XRD confirms the formation of the perovskite phase from ball-milling of precursors. Furthermore, the high-resolution signals presented in Fig. 1b, c and e reveal a shift towards higher angles (smaller interatomic distances) when CsBr is partly replaced by KBr. This means that K^+^, which has a smaller ionic radius than Cs^+^, is effectively incorporated in the perovskite crystal structure, leading to mixed-cation (KCs)PbBr_3_. Such a cation-replacement is not trivial, as potassium is thought to be too small to occupy the “A” site in APbBr_3_.^28,30,31^ Indeed, concomitant to the shift of the main perovskite peaks, we also note that new peaks appear in the diffractogram (see Fig. S1† and 1a, d, f). These peaks are consistent with the non-perovskite APb_2_Br_5_ phase. For potassium-based lead halide compounds, this phase is the most commonly reported.^35,36^ We also ball-milled pure KBr and PbBr_2_ mixtures (without CsBr) in different ratios and found that KPb_2_Br_5_ was the dominant phase – along with unreacted KBr – even in KBr-rich conditions, see Fig. S2.† Therefore, we can conclude that the use of KBr as a source of K^+^ to replace Cs^+^ in inorganic perovskites is possible but limited by the higher stability of KPb_2_Br_5_ as compared to KPbBr_3_. When we reduced the amount of KBr to 5% (A = K_0.05_Cs_0.95_) we also observed similar perovskite peak shifts and formation of KPb_2_Br_5_, although to a lesser extent (see Fig. S3†). This suggests that the amount of Cs^+^ that can be replaced by K^+^ in the perovskite structure (without leading to the formation of KPb_2_Br_5_) is below 5%. This value is lower than previously reported by others.^29^ Other characterization methods such as high-resolution transmission electron microscopy and energy dispersive X-ray spectroscopy could possibly further elucidate the amount of potassium that is present under each form (included in the perovskite lattice or as separated KPb_2_Br_5_ compound). The optical characterization of the powders resulting from ball-milling equimolar ABr:PbBr_2_ mixtures with A = K_0.2_Cs_0.8_ is presented in Fig. 1g–i. Absorption (g) and photoluminescence (h) spectra are mostly unchanged with respect to the reference sample (A = Cs). Photoluminescence spectra (Fig. 1h) evidently consist of two sub-bands with maxima at about 522 and 540 nm (see deconvolution of the PL spectra as a sum of two Gaussian contours in ESI, Fig. S4†). Because of the broad and asymmetric nature of the PL spectra, it is not possible to unambiguously evaluate the impact of potassium incorporation on the optical bandgap. Indeed, it could be expected that the observed shrinkage of the lattice would affect the optical bandgap of the material and result in a shift of the PL peak. However, the origin of the two bands observed in both PL spectra might be due to different reasons. In a previous report on mechanosynthesis of CsPbBr_3_*via* ball-milling of CsBr and PbBr_2_ a similar asymmetric spectrum was obtained and attributed to the presence of bulk and nano-sized CsPbBr_3_.^16^ Another possible explanation is linked to the emission from free electrons in conducting band and trap-localized carriers.^37^ In this second hypothesis, it is possible that an exchange of a part of Cs atoms by K decreases the relative contribution of the PL emission from free electrons at 522 nm and, respectively, increases contribution of the emission from trap states at 540 nm. Following the delayed luminescence model,^38^ trap-assisted luminescence should be longer lived than the emission of free electrons from the conducting band, as is indeed observed in Fig. 1i. However, we cannot exclude other possible origins of this longer lifetime such as trap passivation by molecular KBr which fills halide vacancies at the surface^28^ or by the other two mechanisms that our data prove to happen concomitantly: (i) replacement of Cs^+^ by K^+^ as monovalent cation in the perovskite structure, and (ii) formation of KPb_2_Br_5_ which might act as passivating layer on top of CsPbBr_3_. This passivation (independently on the exact mechanism from which it originates) should result in a higher photoluminescence quantum yield (PLQY). However, the absolute PLQY of these powder samples is too low for us to conduct reliable measurements.

**Fig. 1:**
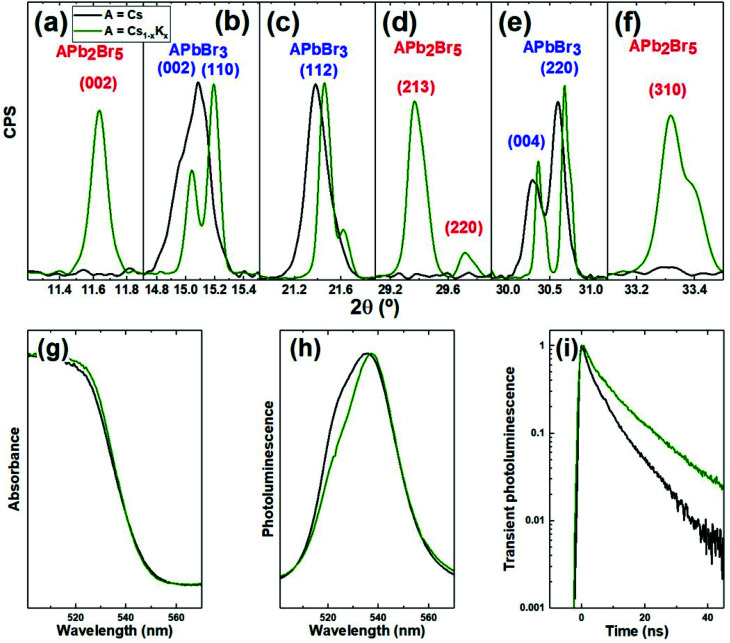
XRD (a–f) and optical (g–i) characterization of powders prepared from addition of PbBr_2_ to CsBr (REF; black lines) or Cs_0.8_K_0.2_Br (green lines). XRD peaks corresponding to APbBr_3_ perovskite (b, c, and e) present a shift upon addition of KBr. Panels (a, d, and f) present a rise in intensity linked to the formation of non-perovskite APb_2_Br_5_ phase. Full diffractograms are presented in Fig. S1.† Absorption (g) and photoluminescence (h) spectra remain mostly unchanged while photoluminescence lifetime (i) is increased.

We also replaced KBr by KX (X = Cl or I) while keeping CsBr and PbBr_2_ as precursors in the mechanosynthesis. Fig. 2 shows XRD and optical characterization of the resulting powders. Fig. 2a–c demonstrate that the perovskite phase is formed in all cases and that the heteroanion (Cl or I) introduced *via* the potassium salt is replacing Br in the APbX_3_ structure. Indeed, when KI is used the main perovskite peaks shift towards lower diffraction angles consistent with the introduction of the larger I^−^ anion compared to Br^−^. The opposite applies when KCl is used. As a result, we observe significant shifts in the bandgap of the perovskite as shown by absorption and photoluminescence (Fig. 2d, e). Hence, our results show that KX can also be used as a source of anions to tune the optical properties of the resulting inorganic perovskite. This means that the X halide does not only remain tightly bound to K^+^ at the surface of the perovskite material affecting only surface-related effects (surface quenching traps) but also enters the structure and thus affects bulk-related properties (bandgap).

**Fig. 2:**
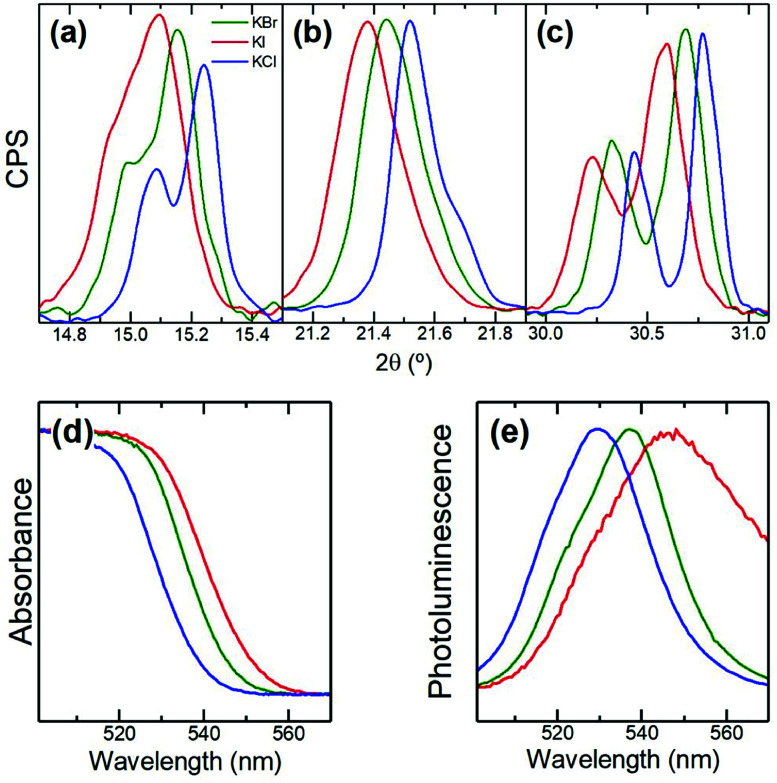
XRD (a–c) and optical (d and e) characterization of powders prepared from KI (red), KBr (green), and KCl (blue). Shifts in diffractograms are consistent with the incorporation of the heteroanion (I or Cl) in the perovskite structure. This translates into a smaller (KI) or higher (KCl) bandgap as observed in absorption (d) and photoluminescence (e).

In conclusion, we have shown that incorporating potassium halides in the mechanosynthesis of inorganic cesium lead halide perovskites leads to several chemical, structural and optical effects. First of all, potassium partly replaces cesium in the APbBr_3_ perovskite structure. Second, the potassium salt can also act as a source of heteroanions to tune the bandgap of the resulting perovskite. Third, KPb_2_X_5_ phase forms concomitantly with the perovskite phase. This phase may act as a surface passivation layer as longer lifetimes are observed on samples with added KBr with respect to pure CsPbBr_3_. These findings will aid to further optimize thin film perovskite based devices such as LEDs and solar cells that recently have shown beneficial effects of incorporating potassium halides.

## Conflicts of interest

There are no conflicts to declare.

## Supplementary Material

RA-008-C8RA08823C-s001
