# Supplementary material for: Incorporation of potassium halides in the mechanosynthesis of inorganic perovskites: feasibility and limitations of ion-replacement and trap passivation
Source: RSC Adv. 2018 Dec 12;8(72):41548–51. doi: 10.1039/c8ra08823c (PMC9091861; doi:10.1039/c8ra08823c)
Supplement: RA-008-C8RA08823C-s001 [file RA-008-C8RA08823C-s001.pdf]

Electronic Supplementary Material (ESI) for RSC Advances.
This journal is © The Royal Society of Chemistry 2018

Supporting information

Incorporation of potassium halides in the mechanosynthesis of inorganic perovskites: feasibility and limitations of ion-replacement and trap passivation

Yousra El Ajjouri,[a] Vladimir S. Chirvony,[a,b] Michele Sessolo,[a] Francisco Palazon*[a] and Henk J. Bolink[a]

[a.] Instituto de Ciencia Molecular, ICMol, Universidad de Valencia, C/ Catedrático J. Beltrán 2, 46980 Paterna, Spain

[b.] UMDO (Unidad de Materiales y Dispositivos Optoelectrónicos), Instituto de Ciencia de los Materiales, Universidad de Valencia, Valencia 46071, Spain

Experimental

Chemicals

Cesium bromide (CsBr, > 99 %), was purchased from TCI. Potassium bromide (KBr, > 99%) and lead(II) bromide ($PbBr_2$, ≥ 98 %) were purchased from Sigma-Aldrich. All chemicals were stored in a nitrogen-filled glovebox and used as received without further purification.

Synthesis

Equimolar AX : $PbBr_2$ powders (AX = CsBr or $CsBr_{0.8}KY_{0.2}$; where Y = I, Br, or Cl) were mixed inside a nitrogen-filled glovebox. Then, approximately 3 grams of the mixed precursors powders were introduced inside 10 mL zirconia ball-mill jars with 2 zirconia beads of 10 mm in diameter. The jars were closed under nitrogen so that the powders were not exposed to air. Then ball-milling was performed with a MM-400 straight ball-mill from Retsch, at a frequency of 30 Hz for 5 hours.

XRD

X-ray diffraction was measured with a Panalytical Empyrean diffractometer equipped with CuKα anode operated at 45 kV and 30 mA and a Pixel 1D detector in scanning line mode. Single scans were acquired in the 2Θ = 10° to 50° range in Bragg-Brentano geometry in air. Data analysis was performed with HighScore Plus software.

Absorbance and photoluminescence spectroscopy

Absorbance was measured with a High Power UV-VIS fiber light source, integrated sphere and Avantes Starline AVASpec-2048L spectrometer in reflection mode. Photoluminescence was

measured with a continuous wave 375 nm diode laser with a 400nm filter, and Hamamatsu PMA 11 spectrometer. For a typical spectrum 10 scans of 1 second were averaged.

Photoluminescence measurements

A third harmonic (355 nm) of a Nd:YAG pulsed laser with 1.1 ns pulse duration, 1 kHz pulse repetition frequency and 20μJ/cm^2 excitation density was used as an excitation source for the PL measurements. To detect the time-resolved PL kinetics an integrated PL signal was directed through a multimode optical fiber to a time correlated single photon counting electronic board. For the PL spectra measurements, the PL signal was directed through an optical fiber to an Ocean type spectrometer.

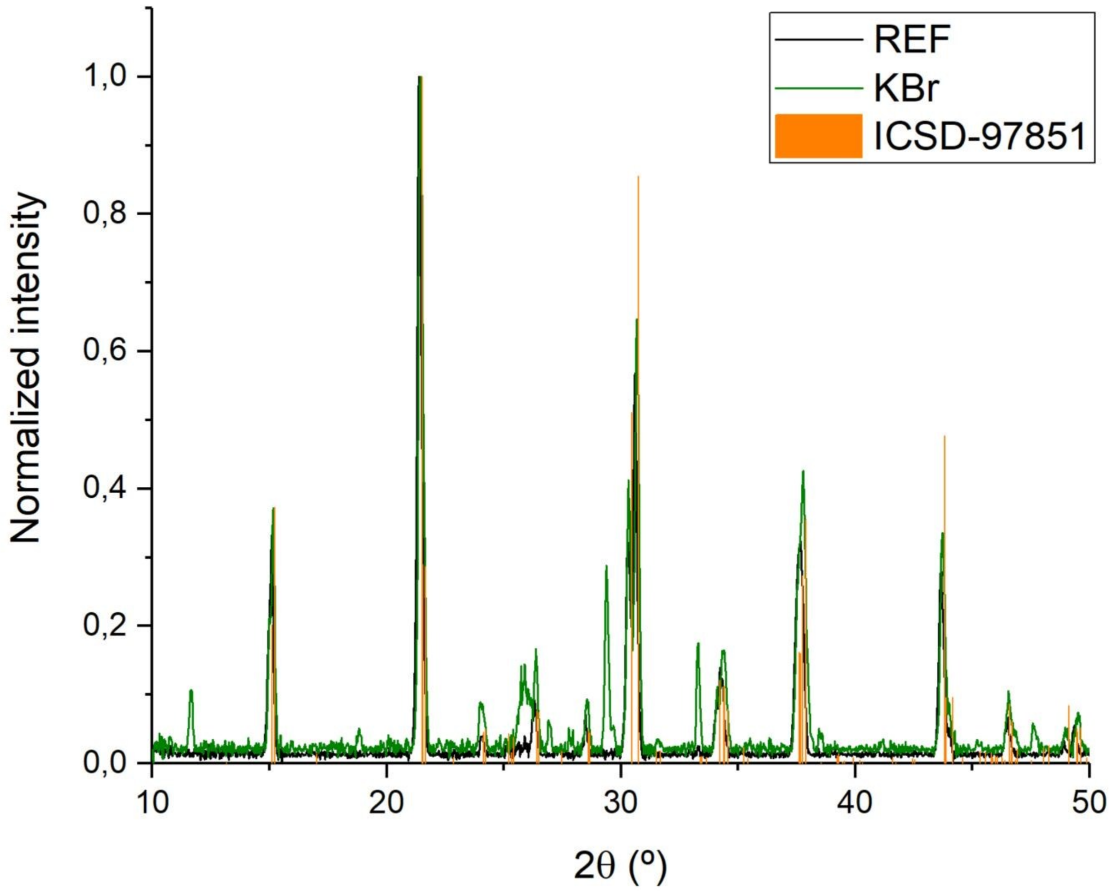

Figure S 1. XRD signal of samples prepared from pure CsBr (black line) and partly (20%) substituted by KBr (green line). Selected areas of the diffractograms are shown in Figure 1. Reference pattern for bulk orthorhombic $CsPbBr_3$ is shown as orange columns.

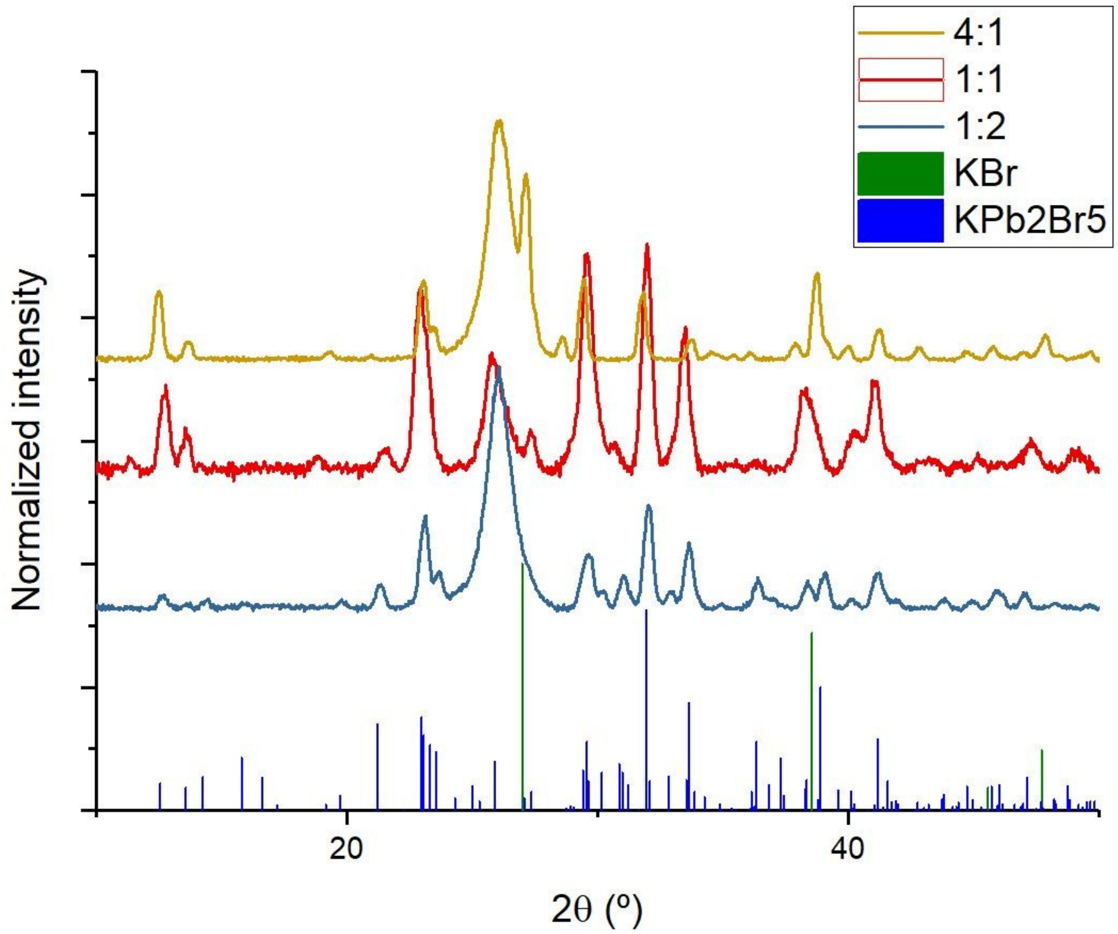

Figure S 2. Diffractograms of powders prepared by ball-milling KBr and $PbBr_2$ in different molar ratios (1:2, 1:1, and 4:1). Reference patterns for bulk KBr (ICSD: 18015) and KPb_2Br_5 (ICSD: 250266) are presented as green and blue columns. The broad signal around 2Θ = 25° corresponds to the substrate on which the powders are deposited for XRD characterization.

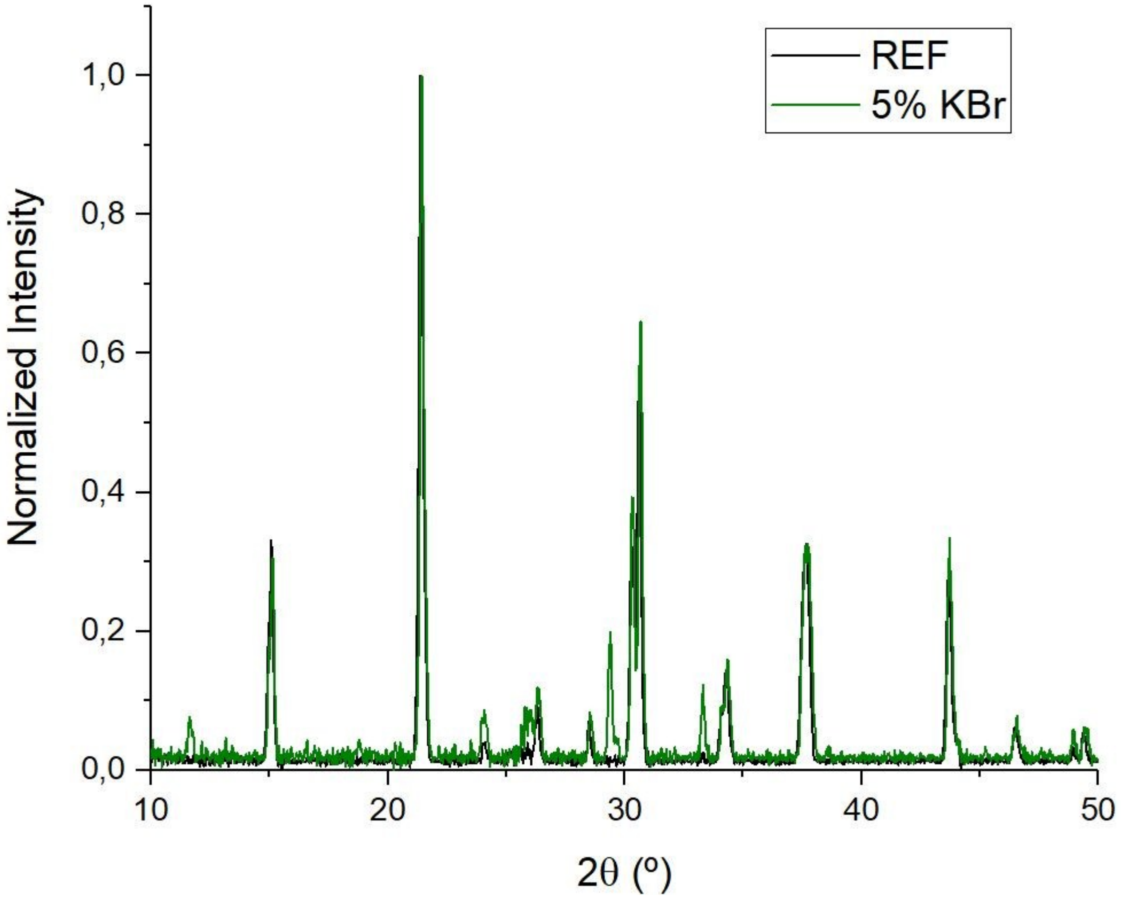

Figure S 3. XRD signal of samples prepared from pure CsBr (black line) and partly (5%) substituted by KBr (green line). Effects of KBr are qualitatively similar than in the case of 20% (Figure S1 and Figure 1) although to a lesser extent, as may be expected by a lower percentage of KBr.

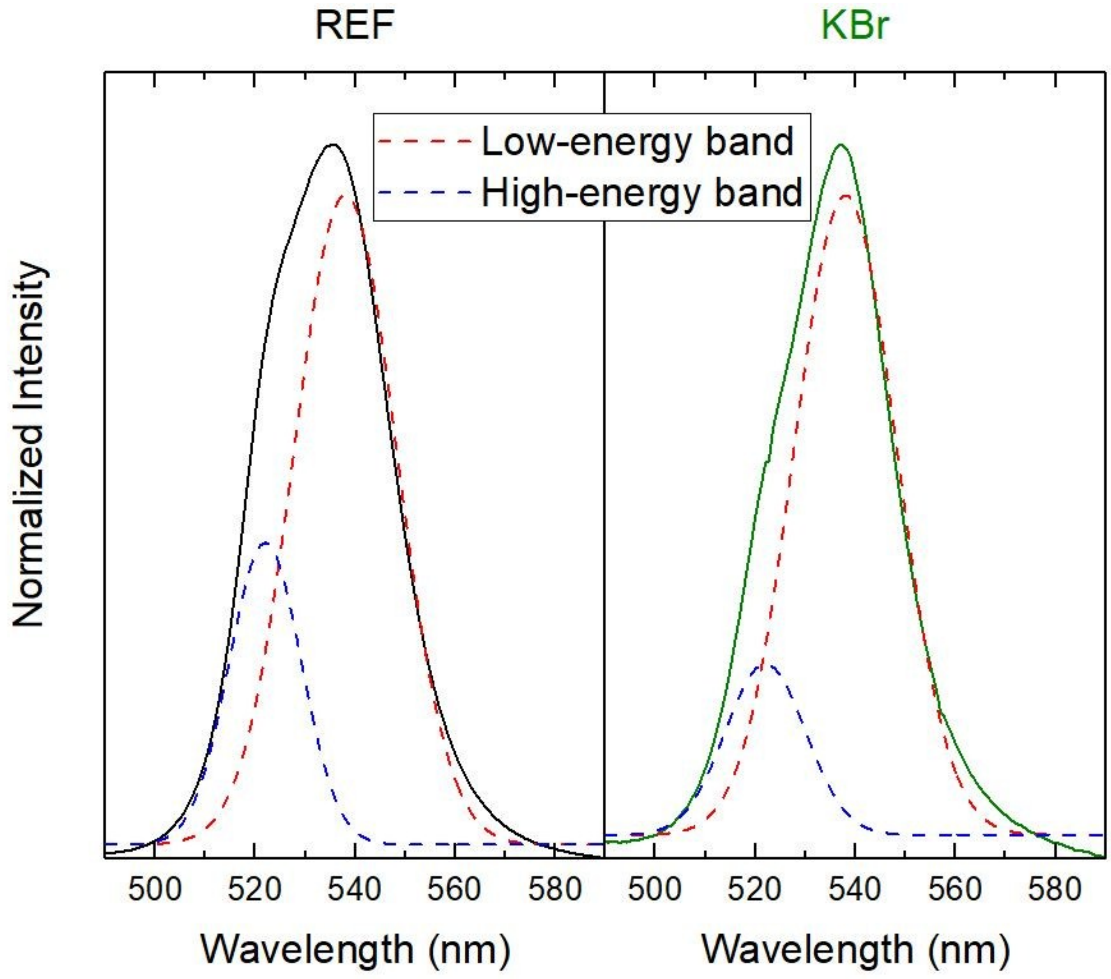

Figure S 4. Photoluminescence spectra of reference and KBr sample fitted with two gaussian peaks. The KBr sample shows a reduction in the relative intensity of the high-energy component as explained in the main text.
